# Evaluating Performance of Transvaginal Doppler Parameters in Differentiating Cervical Neoplastic Severity

**DOI:** 10.3390/diagnostics16162592

**Published:** 2026-08-16

**Authors:** Tuğçe Sırma, Konul Mehdiyeva, Gürdeniz Serin, Halil İbrahim Tiraş, Mert Acar, Ahmet Aydın Özsaran, Mustafa Coşan Terek, Levent Akman, Nuri Yıldırım

**Affiliations:** 1 Department of Gynecological Oncology Surgery, Iskenderun State Hospital, Hatay 31200, Turkey; drtugcesirma@hotmail.com; 2 Department of Obstetrics and Gynecology, Kirklareli Training and Research Hospital, Kırklareli 39000, Turkey; konulmehdiyeva@hotmail.com; 3 Department of Pathology, Faculty of Medicine, Ege University, Izmir 35100, Turkey; 4 Department of Gynecological Oncology Surgery, Faculty of Medicine, Ege University, Izmir 35100, Turkey; ibbrahim.iz@gmail.com (H.İ.T.); ozsarana@gmail.com (A.A.Ö.); terekmc@yahoo.com (M.C.T.); leventakman@gmail.com (L.A.); 5 Department of Obstetrics and Gynecology, Nusaybin State Hospital, Mardin 47300, Turkey; drmertacar@gmail.com

**Keywords:** uterine artery, cervical intraepithelial neoplasia, cervical cancer, Doppler parameters

## Abstract

**Background/Objectives**: Angiogenesis plays a central role in the progression of cervical intraepithelial neoplasia (CIN) to cervical cancer (CC). Transvaginal Doppler ultrasonography (TVDUSG) offers a non-invasive means of assessing hemodynamic changes associated with neoplastic transformation. This study aimed to evaluate the diagnostic performance of uterine artery (UA) Doppler parameters across the full cervical disease spectrum and their associations with adverse prognostic features and clinicopathological features in CC. **Methods**: This prospective observational cohort study enrolled 170 patients who were divided into four groups: controls, CIN 1, CIN 2–3, and CC. All underwent TVDUSG prior to treatment. Pulsatility index (PI), resistance index (RI), peak systolic velocity (PS), end-diastolic velocity (ED), PS/ED ratio, ED/PS ratio, and time-averaged maximum velocity were recorded. ROC curve analysis was performed to assess diagnostic performance. **Results**: PI and RI increased progressively from controls to CC, while ED declined across groups (all *p* < 0.001). In patients with CC, PI correlated with tumor volume and was elevated in those with advanced-stage, parametrial and lymph node involvement (all *p* < 0.001). ROC analysis revealed strong diagnostic performance for PI in distinguishing CC from controls (AUC = 0.861, sensitivity 80.0%, specificity 80.4%); however, PI showed limited ability to distinguish cervical cancer specifically from high-grade CIN (AUC = 0.593). **Conclusions**: UA Doppler parameters, particularly PI, are associated with cervical neoplastic severity and adverse prognostic features in CC. TVDUSG may serve as a practical, non-invasive adjunct in the preoperative evaluation of cervical neoplasia, especially where advanced imaging is unavailable.

## 1. Introduction

Cervical cancer (CC) ranks as the fourth most common malignancy among women globally, representing about 8.0% of all female cancer-related deaths every year [1,2,3]. Despite a reduction in both incidence and mortality resulting from the implementation of the human papillomavirus (HPV) screening programs and vaccination initiatives, CC remains a global health issue, especially in developing countries [4]. GLOBOCAN 2022 reported a total of 661,021 new cases of CC and 348,189 deaths worldwide in that year [5]. High-grade cervical intraepithelial neoplasia (CIN), particularly CIN 3, is regarded as a precancerous lesion with an estimated risk of progression to invasive cervical cancer of up to 30% over a 30-year period [6]. Persistent infection with high-risk human papillomavirus (hr-HPV) is the primary etiological factor for CC and precancerous lesions [7].

Currently, the essential diagnostic modalities for CC and precancerous lesions include cervical cancer screening programs, such as the Pap-smear test and HPV testing, as well as colposcopy and cervical biopsy. Before 2018, clinical evaluation was based solely on clinical evaluation for the International Federation of Gynecology and Obstetrics (FIGO) cervical cancer staging system, which was incorporated with imaging and pathology assessment afterwards [1,8].

Angiogenesis is the production of new vessels in a specific region and is required for tumor growth and progression, and correlates with tumor metastatic potential [9]. Several investigations have shown that angiogenesis and vascularity in CC are associated with specific tumor features and prognostic parameters for relapse [10,11,12,13,14]. The vascular structure of the tumor is distinct from that of the normal tissue [15]. The two defining signs are arteriovenous shunts and newly formed big capillaries or sinusoids lacking smooth muscle in their walls, both typical characteristics of neoangiogenesis, leading to high flow velocity and low resistance, respectively [15,16]. It has been shown that the progression of the lesion from CIN to CC is accompanied by angiogenesis [9,17]. As a result, research has increasingly concentrated on employing ultrasonography to examine blood flow, vascular distribution, and the evolution of cervical lesions, with the objective of differentiating between CIN and CC [2]. Moreover, previous investigations have demonstrated that local cervical blood vessel counts and vascular volumes significantly increased in high-grade CIN compared with the cervicitis; also, perfusion patterns revealed a gradual enhancement with increasing CIN grade [18].

Traditionally, angiogenesis in tumors is assessed by immunohistochemical staining, but with the application of ultrasound Doppler imaging, tumor vascularity and therefore the process of angiogenesis can be observed in vivo [19]. Transvaginal Ultrasonography (TVUSG) is a non-invasive and attractive modality because it is cost-effective, faster, more widely available and can be performed repeatedly without ionizing radiation exposure and requires no preparation of the patient [19,20]. Color Doppler (CD) is commonly utilized to assess the visualization of vessels by color-coding for angiogenesis in precancerous and cancerous lesions [2]. Power Doppler (PD) is an effective mapping technique for assessing tumor blood flow, characterized by its independent relative angle and velocity, an enlarged dynamic range, and enhanced sensitivity [2,21]. However, the clinical value of changes in Doppler parameters in pre-malignant lesions and CC is still uncertain.

Therefore, the current study aimed to investigate the usefulness of transvaginal Doppler parameters in evaluating uterine artery (UA) blood flow in order to elucidate their association with pre-malignant lesions and CC compared with healthy controls.

## 2. Materials and Methods

### 2.1. Study Design and Clinical Information

This prospective observational cohort study enrolled 170 patients at the Department of Gynecological Oncology of Ege University, İzmir. The study was conducted over a period of 7 months (from March 2025 to October 2025) following approval from the Institutional Ethics Committee of Ege University Hospital (Approval Number: 25-2.1/10) and was conducted in accordance with the Declaration of Helsinki. Informed consent was obtained from all women after a full explanation of the objectives of the study.

The study population comprised patients with histologically proven CC or CIN. The control group was defined as having normal cytology result, normal HPV result, normal TVUSG of the cervix, uterus, and adnexes, no symptoms, and no pathological findings of the genital organs on pelvic examination [10]. For the 15 patients treated with primary chemoradiotherapy, lymph node status was assigned based on preoperative PET-CT findings and, where applicable, incorporated into FIGO 2018 nodal staging (stage IIIC1/IIIC2); for surgically treated patients, nodal status was based on pathological evaluation following pelvic lymphadenectomy [2]. For this study, we collected demographic and clinical data about the patients, including age, hr-HPV infection, menopausal status, smoking, symptoms, gravidity and parity, and primary treatment [22].

All patients underwent a transvaginal Doppler ultrasonography (TVDUSG) prior to treatment including cryotherapy, cervical excisional procedure (loop electrosurgical excision procedure (LEEP)/conization), primary surgery or primary chemoradiotherapy (CRT). All patients with an initial diagnosis of CIN 2-3 underwent LEEP. The inclusion criteria were as follows: 1—no history of other malignant tumors; 2—complete clinical data; 3—no history of cervical surgery or embolization of the uterine arteries; 4—patients with newly biopsy detected squamous cell carcinoma (SCC), adenosquamous carcinoma or adenocarcinoma, and CIN; 5—no synchronous or metastatic cancer; 6—patients >25 and <65 years; 7—no serious damage to cardiovascular, liver, and kidney function, and complete blood count and coagulation parameters [23].

Our exclusion criteria included patients who did not meet the aforementioned inclusion criteria, in addition to patients who had previously undergone hysterectomy, those with a history of intravaginal drug use, intrauterine device or oral contraceptive use, and other unrelated clinical conditions, such as cervical myomas, significant Nabothian cysts, and various cervical masses, any uterine disease, for example myomas, adenomyosis, endometrial hyperplasia, and any adnexal masses [10,24]. Patients with abnormal uterine bleeding, congenital malformations of the genital tract, diabetes, genital infections, such as cervicitis, pelvic inflammatory disease (PID), and vaginitis, cervical stump cancer, or those who were in menstrual, gestation or lactation period were also excluded [7,11,19].

Clinical staging for the 20 patients undergoing primary surgery was based on examination under general anesthesia combined with preoperative PET/CT in all cases; pelvic contrast-enhanced MRI was additionally performed when parametrial involvement was clinically suspected.

### 2.2. Ultrasonography Examination

All transvaginal Doppler examinations were performed by a single examiner (T.S., 10 years of experience) skilled in gynecological oncology to avoid interobserver and intraobserver variability using a Voluson E8 ultrasound system (GE Healthcare, Zipf, Austria) equipped with a 5–9 MHz endovaginal probe.

#### 2.2.1. Doppler Measurement Procedure

Each patient was prepared for the examination by emptying their bladder and being placed in the lithotomy position on the examination bed [25]. The ultrasound examination commenced with routine disinfection of the external and internal genitalia, after which the vaginal probe was covered with a disposable condom and a coupling gel was applied [7,26]. The probe was then gently inserted into the vagina until it reached the vaginal fornix [26]. The structure of the genital organs, emphasizing the shape and echogenicity of the cervix-parametrial tissues, and the pelvic cavity were thoroughly evaluated with 2D-grayscale ultrasound to determine whether any lesions were present [25]. Tumor echogenicity was categorized as hypoechoic, isoechoic, hyperechoic, or mixed, with irregular boundaries in relation to the adjacent cervical tissue [27].

**Figure 1:**
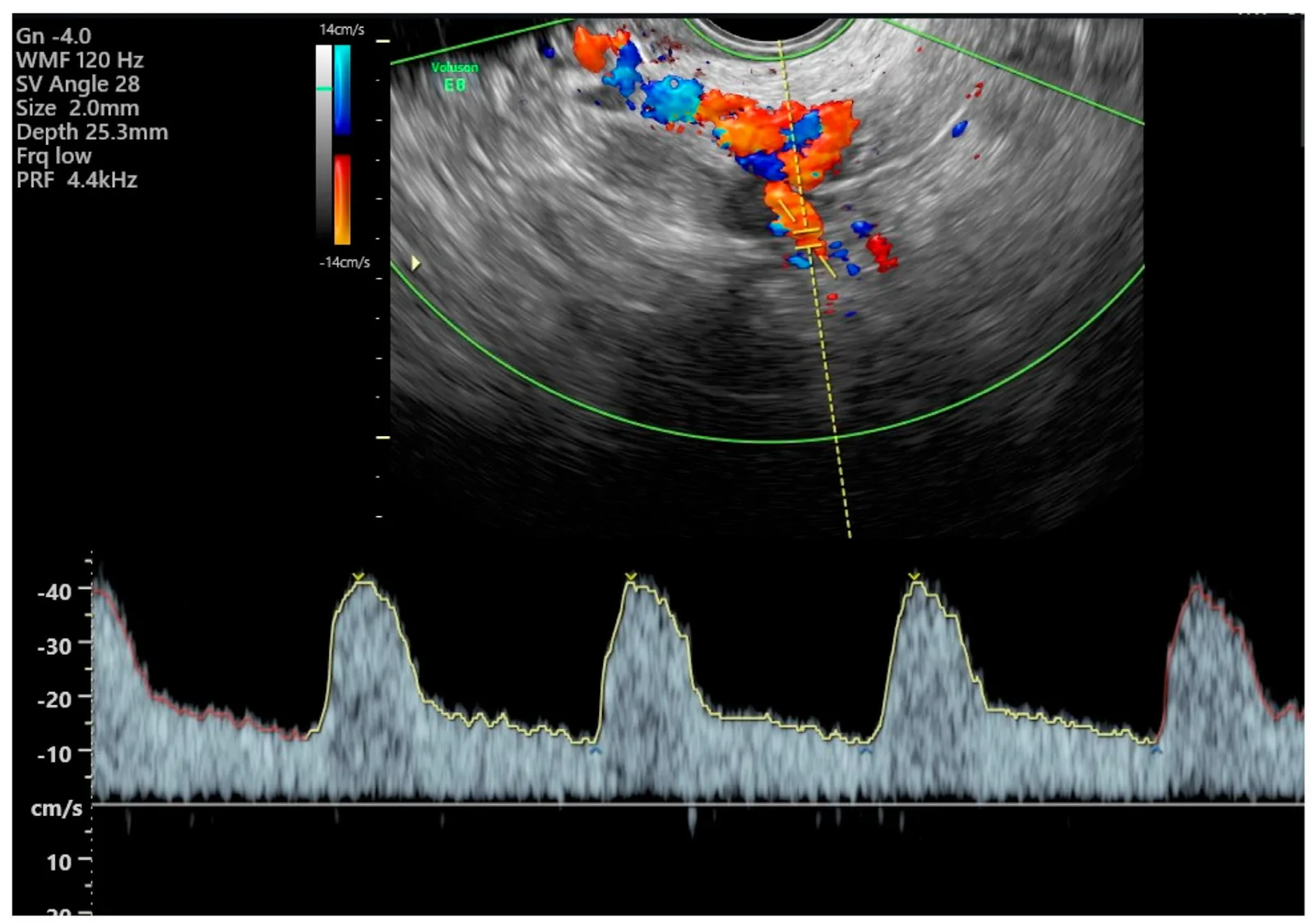
The Power Doppler examination of the uterine artery in a 47 year old patient diagnosed with FIGO stage IB2 mucinous adenocarcinoma of cervix.

Color Doppler was activated in all cases after morphological evaluation to evaluate the distribution of blood flow signals inside and around the cervical area [8,9]. The probe was further repositioned laterally until the paracervical vascular plexus appeared, and the UA was located at the isthmus level in the parasagittal plane [24]. Power Doppler and pulsed-wave Doppler flow mapping were carried out at this site prior to the artery branching into the ascending and descending arteries [28]. Detectable Doppler signals were described as a sequence of repeatable, analogous arterial waveforms acquired across a minimum of three distinct, consecutive cardiac cycles [29]. Blood flow signals were measured from the unilateral UA (Figure 1). The same process was repeated on the contralateral side if the vessel was not identified clearly on one side [16]. All parameters were obtained automatically, including peak systolic velocity (PS), end-diastolic velocity (ED), time-averaged maximum velocity (TAMAX), pulsatility index (PI), and resistance index (RI) [29]. The PI was described as the ratio of the difference between PS and ED to mean velocity [29]. The RI was defined as the ratio of the difference between the PS and ED to PS [29].

#### 2.2.2. Sample Size Determination and Bias Reduction Strategies

Sample size was calculated a priori using G*Power software (version 3.1.9.7; Heinrich Heine University, Düsseldorf, Germany), based on a two-sample independent *t*-test framework. At the design stage, no published data on uterine artery Doppler indices across the cervical neoplastic spectrum were available for an a priori sample size calculation. Reference variance estimates were therefore derived from intratumoral pulsatility index values reported in a related cervical cancer Doppler study [30] comparing patients with and without lymph node metastasis (PI 0.25 ± 0.06 vs. 0.31 ± 0.08), which is not directly comparable to uterine-artery-level measurements. Based on these values, a minimum of 31 patients per group (124 in total) was calculated to provide 90% power at a two-sided α of 0.05; the final enrollment of 170 patients exceeded this a priori threshold. Given the limited comparability of the original reference source, achieved power was additionally recalculated post hoc using the observed uterine artery PI values in the present cohort (control: 1.49 ± 0.54; cervical cancer: 2.38 ± 0.68), yielding an effect size (Cohen’s d) of 1.45 and an achieved power exceeding 99% at the enrolled sample size. To minimize systematic bias, all Doppler measurements were performed by a single experienced examiner blinded to the final histopathological diagnosis at the time of ultrasonographic evaluation. Patient allocation to study groups was based exclusively on histopathological results obtained after ultrasonographic assessment.

### 2.3. Statistical Analysis

All statistical analyses were performed using the Statistical Package for the Social Sciences (SPSS) version 27.0 (IBM Corp., Armonk, NY, USA). Descriptive statistics were presented as mean ± standard deviation for normally distributed continuous variables, median with interquartile range (IQR) for non-normally distributed continuous variables, and frequencies with percentages for categorical variables. The normality of data distribution was assessed using the Shapiro–Wilk test and visual inspection of histograms and Q-Q plots. For comparisons among multiple groups (control, CIN 1, CIN 2-3, and cervical cancer), one-way analysis of variance (ANOVA) was used for normally distributed continuous variables, while the Kruskal–Wallis test was applied for non-normally distributed continuous variables. Categorical variables were compared using the Chi-square test or Fisher’s exact test when the expected cell frequency was less than 5. Post hoc pairwise comparisons were performed with Bonferroni correction when significant differences were detected among groups. For comparisons between two independent groups (such as presence vs. absence of parametrial involvement, lymphovascular space invasion [LVSI]), or lymph node [LN] metastasis), the Mann–Whitney U test was used for continuous variables based on the non-normal distribution of Doppler parameters. Correlations between continuous variables (such as Standardized Uptake Value maximum [SUVmax], depth of stromal invasion, tumor volume, and Doppler parameters) were assessed using Spearman’s rank correlation coefficient due to the non-parametric nature of the data. Receiver operating characteristic (ROC) curve analysis was performed to evaluate the diagnostic performance of Doppler parameters in discriminating cervical cancer from healthy controls and from CIN. The area under the curve (AUC) with 95% confidence intervals was calculated for each parameter. Optimal cutoff values were determined using the Youden index (sensitivity + specificity − 1). A *p*-value of less than 0.05 was considered statistically significant. To assess potential confounding by age and menopausal status, multivariable linear regression models were constructed with PI and RI as dependent variables and disease group, age, and menopausal status as covariates; univariable associations were also reported for comparison, and a sensitivity analysis restricted to premenopausal patients was performed. False discovery rate correction (Benjamini–Hochberg method) was applied to account for multiple testing. Internal validation of ROC-derived cutoffs was performed using bootstrap resampling (2000 iterations) to obtain optimism-corrected sensitivity and specificity estimates. For each bootstrap resample, the Youden-optimal cutoff was re-derived independently and applied to the original sample to obtain optimism-corrected sensitivity and specificity estimates; the AUC itself, being a rank-based statistic not dependent on cutoff selection, was not subject to the same correction. False discovery rate correction was additionally applied to the 28 comparisons, using the same Benjamini–-Hochberg method described above. As a sensitivity analysis, smoking status was additionally included as a covariate in the multivariable models; given the ordinal coding of disease group in the primary model, an indicator (dummy) variable coding was also examined, together with variance inflation factor (VIF) diagnostics to assess collinearity.

## 3. Results

Table 1 reports the clinical characteristics of the whole population divided into the four groups. One hundred and seventy women were enrolled in the study, comprising 46 patients in the control group, with patients categorized into CIN 1 (*n* = 43), CIN 2-3 (*n* = 46), and cervical cancer (*n* = 35) groups. The mean age increased progressively from the control group (38.50 ± 7.41 years) to the cervical cancer group (48.02 ± 12.79 years, *p* = 0.001). The majority of the patients were premenopausal (*n* = 131) demonstrating significant differences among groups, with postmenopausal women comprising 13.04% of controls compared to 45.71% of cervical cancer patients (*p* = 0.002). A statistically significant difference was observed between groups with respect to parity (*p* = 0.015), whereas gravidity did not reach statistical significance (*p* = 0.065) particularly when compared with the CIN 2–3 group. On the other hand, type of delivery was found to be non-significant (*p* = 0.099). A high proportion of patients with cervical cancer (*n* = 25) had symptoms. Primary chemoradiotherapy was administered to 15 patients with cervical cancer (42.86%).

HPV status was based on documented negative screening history within the preceding year for asymptomatic patients with normal cytology (26 of 46 controls) or on current negative HPV results before Doppler evaluation (remaining patients). Missing values in the CIN and cervical cancer groups largely reflect patients presenting with abnormal cytology or clinical symptoms warranting colposcopy without a prior HPV test on record, most notably among cervical cancer patients who had not previously undergone routine screening.

A statistically significant difference was detected in the UA Doppler values according to the severity of cervical lesions (Table 2). From the control (1.49 ± 0.54) and CIN 1 (1.68 ± 0.42) groups to the CIN 2–3 (2.14 ± 0.57) and cervical cancer (2.38 ± 0.68) groups, the PI demonstrated a stepwise increase, with substantially higher values in the latter (*p* = 0.001). A similar pattern was found for RI, increasing progressively from 0.74 (0.64–0.79) in controls to 0.84 (0.80–0.89) in cervical cancer patients (*p* = 0.001).

In contrast, PS measurements ranged from 36 (24–49) cm/s in controls to 41 (28–53) cm/s in cervical cancer cases, though these differences did not reach statistical significance (*p* = 0.096). Similarly, TAMAX did not differ significantly among groups (*p* = 0.797).

On the other hand, ED demonstrated a statistically significant progressive decline with increasing disease severity, with the cervical cancer group exhibiting the lowest values (CC: 5 (4–8) cm/s vs. control: 10 (7–13) cm/s) (*p* = 0.001). It was observed that a low ED value resulted in the significantly higher PS/ED ratio (*p* = 0.001) and a lower ED/PS ratio (*p* = 0.001) in the cervical cancer and CIN 2-3 groups in comparison to the control group and CIN 1.

Doppler parameters were assessed in connection to final histopathology results in patients who underwent LEEP for high-grade CIN (Table 3). Doppler parameters did not effectively differentiate between those with confirmed high-grade disease (CIN 2–3) and low-grade (CIN 1) or no disease following LEEP in patients with an initial CIN 2 diagnosis, as no significant differences were noted across all assessed indices (PI, RI, PS, ED, PS/ED, ED/PS, and TAMAX; all *p* > 0.05). Likewise, lesion volume exhibited no significant correlation with any Doppler parameter (all *p* > 0.05).

On the contrary, in patients initially diagnosed with CIN 3, the PI was nominally higher in those with confirmed high-grade disease (CIN 2–3) compared to those with confirmed CIN 1 or none following LEEP (2.2 [1.9–2.6] vs. 1.4 [1.2–1.9], respectively, *p* = 0.042); however, this association did not remain significant after false discovery rate correction (*p* = 0.266) and should be regarded as exploratory given the small comparator group (*n* = 5). Lesion volume demonstrated nominally significant positive correlations with PI (r = 0.499, *p* = 0.011), RI (r = 0.410, *p* = 0.042), and PS/ED (r = 0.406, *p* = 0.044), along with a nominally significant negative correlation with ED/PS (r = −0.400, *p* = 0.047); none of these correlations remained significant after correction for multiple testing. There were no significant relationships with PS, ED, or TAMAX (all *p* > 0.05).

According to FIGO 2018 criteria, significant connections were found between Doppler indices and clinicopathological characteristics of cervical cancer (Table 4). The SUVmax for F-18 fluorodeoxyglucose exhibited a notable positive association with PI (r = 0.583, *p* = 0.001) and a significant negative correlation with ED/PS (r = −0.358, *p* = 0.035), whereas no significant relationships were observed with the other Doppler parameters (all *p* > 0.05).

The tumor volume demonstrated significant positive associations with RI (r = 0.446, *p* = 0.007), PI (r = 0.741, *p* = 0.001) and a significant inverse correlation with ED/PS (r = −0.422, *p* = 0.012), while no statistically significant correlations were identified with PS, ED, PS/ED, or TAMAX (all *p* > 0.05). The presence versus absence of LVSI was not significantly associated with PI (2.0 [1.8–2.2] vs. 1.9 [1.6–2.2], *p* = 0.670); however, LVSI positivity was significantly associated with RI (*p* = 0.047). Other Doppler parameters exhibited no statistically significant changes (all *p* > 0.05). Nominal disparities in PI and TAMAX were noted between histologic subtypes (SCC vs. non-SCC, both *p* = 0.021), though neither remained significant after correction for multiple testing. Nevertheless, there were no statistically significant relationships between the depth of stromal invasion and any Doppler parameter (all *p* > 0.05).

All patients with a preoperatively clinically evaluated FIGO stage I underwent Wertheim’s hysterectomy with pelvic lymph node dissection (*n* = 20, 57.14%). According to FIGO 2018 criteria, early-stage disease was confirmed in 19 patients (54.2%). The detailed distribution was as follows: stage IA in four patients (11.4%), stage IB in 15 (42.8%), stage IIB in five patients (14.2%), stage IIIC1 in three patients (8.5%), stage IIIC2 in four patients (11.4%), stage IVA in one patient (2.8) and stage IVB in three patients (8.5%). Nine women had a non-SCC histologic type on final pathology.

Another notable result concerned high-risk prognostic factors such as parametrial and lymph node involvement. The presence of parametrial involvement was associated with significantly elevated PI values (3.0 [2.4–3.5] vs. 2.0 [1.7–2.2], *p* = 0.001), PS/ED ratio (7.8 [5.8–11.0] vs. 5.4 [4.7–8.3], *p* = 0.030), and statistically notable RI value and ED/PS ratio (*p* = 0.030 and *p* = 0.046, respectively) compared to cases without parametrial involvement. A similar trend was detected in the LN involvement group, where PI values were 3.0 [2.4–3.5] compared to 2.1 [1.8–2.4] (*p* = 0.001), indicating considerably higher PI levels. We divided our patients with cervical cancer into two groups for the purpose of case comparison: stage I/IIA as an early case and stage IIB/IV as an advanced case as per the FIGO staging; it was found that a significant higher PI among patients with advanced-stage compared with patients with early-stage disease (*p* = 0.001).

**Table 3:** Doppler ultrasound parameters and lesion volume in relation to final histopathological diagnosis after LEEP.

	*n*	PI	RI	PS (cm/s)	ED (cm/s)	PS/ED	ED/PS	TAMAX (cm/s)
Initially CIN 2 Diagnosis underwent LEEP								
CIN 2-3	11	2.1 (1.5–2.4)	0.9 (0.8–0.9)	33.3 (25.1–39.3)	6.0 (3.4–7.8)	6.6 (5.0–7.9)	0.2 (0.1–0.2)	12.9 (10.4–17.2)
CIN 1 or none	10	2.1 (1.9–2.8)	0.8 (0.8–0.9)	45.5 (30.3–54.9)	6.6 (3.4–13.4)	5.6 (4.6–9.6)	0.2 (0.1–0.2)	15.7 (12.4–29.7)
*p*		0.314	0.809	0.223	0.468	1.000	1.000	0.387
Lesion volume (mm)								
r		0.305	0.172	0.218	0.092	0.104	−0.083	0.139
*p*		0.179	0.457	0.343	0.691	0.653	0.720	0.547
Initially CIN 3 Diagnosis underwent LEEP								
CIN 2-3	20	2.2 (1.9–2.6)	0.8 (0.7–0.9)	35.4 (22.8–51.4)	5.5 (4.5–9.0)	5.7 (4.2–6.7)	0.2 (0.2–0.3)	12.7 (8.3–15.5)
CIN 1 or none	5	1.4 (1.2–1.9)	0.8 (0.7–0.8)	39.9 (33.6–51.9)	9.8 (5.0–12.4)	4.2 (3.4–5.9)	0.2 (0.2–0.3)	19.6 (14.3–27.8)
*p*		0.042	0.336	0.621	0.336	0.371	0.272	0.129
Lesion volume (mm)								
r		0.499	0.410	0.046	−0.126	0.406	−0.400	−0.105
*p*		0.011	0.042	0.827	0.549	0.044	0.047	0.618

CIN: cervical intraepithelial neoplasia; LEEP: loop electrosurgical excision procedure; PI: pulsatility index; RI: resistance index; PS: peak systolic velocity; ED: end-diastolic velocity; TAMAX: time-averaged maximum velocity. Following Benjamini–Hochberg correction for multiple testing across the 28 comparisons in this table, none of the associations remained statistically significant; given the small comparator subgroup (*n* = 5 in the CIN 3-initiated cohort), these findings should be interpreted as exploratory and hypothesis-generating rather than confirmatory.

**Table 4:** Correlation and comparison of Doppler parameters with clinical features in patients with cervical cancer.

Clinical Features		*n*	PI	RI	PS (cm/s)	ED (cm/s)	PS/ED	ED/PS	TAMAX (cm/s)
SUVmax	r	35	0.583	0.298	0.129	−0.129	0.066	−0.358	−0.125
	*p*		0.001	0.082	0.462	0.462	0.707	0.035	0.474
Depth of stromal invasion	r	20	0.122	0.010	−0.142	−0.154	0.055	−0.024	−0.196
	*p*		0.609	0.967	0.550	0.516	0.817	0.921	0.408
Tumor volume (mm)	r	35	0.741	0.446	0.292	−0.103	0.275	−0.422	−0.059
	*p*		0.001	0.007	0.089	0.555	0.110	0.012	0.738
Parametrial involvement	Absent	20	2.0 (1.7–2.2)	0.8 (0.8–0.9)	39.4 (28.3–51.0)	6.3 (3.9–8.1)	5.4 (4.7–8.3)	0.1 (0.1–0.2)	15.5 (10.7–24.0)
	Present	15	3.0 (2.4–3.5)	0.9 (0.8–0.9)	42.6 (26.8–55.7)	4.5 (3.5–6.2)	7.8 (5.8–11.0)	0.1 (0.1–0.2)	12.9 (8.6–15.5)
	*p*		0.001	0.030	0.587	0.298	0.030	0.046	0.214
LN involvement	Absent	23	2.1 (1.8–2.4)	0.8 (0.8–0.9)	39.5 (28.0–52.8)	6.2 (3.9–8.4)	5.7 (4.9–8.7)	0.1 (0.1–0.2)	15.5 (10.6–24.0)
	Present	12	3.0 (2.4–3.5)	0.8 (0.8–0.9)	42.3 (29.2–52.8)	4.5 (3.7–5.8)	8.6 (6.1–11.1)	0.1 (0.1–0.2)	12.8 (8.8–13.9)
	*p*		0.001	0.073	0.878	0.184	0.079	0.092	0.132
Stage	Early		2.0 (1.6–2.2)	0.8 (0.8–0.9)	39.5 (28.5–52.9)	6.9 (3.9–8.4)	5.5 (4.8–8.7)	0.2 (0.1–0.2)	17.3 (10.7–24.0)
	Advanced		3.0 (2.3–3.5)	0.8 (0.8–0.9)	42.3 (25.3–54.7)	4.4 (3.7–6.2)	7.5 (5.7–10.5)	0.1 (0.1–0.2)	12.8 (8.2–14.9)
	*p*		0.001	0.102	1.000	0.182	0.109	0.151	0.071
Histologic type	SCC	26	2.4 (2.0–3.0)	0.9 (0.8–0.9)	40.0 (27.1–51.2)	5.0 (3.9–6.2)	7.4 (5.4–9.4)	0.1 (0.1–0.2)	12.8 (9.9–16.9)
	Non-SCC	9	2.1 (1.6–2.2)	0.8 (0.8–0.9)	49.0 (37.3–52.9)	7.7 (6.9–9.5)	5.2 (4.8–7.4)	0.2 (0.1–0.2)	24.0 (13.7–25.4)
	*p*		0.021	0.636	0.299	0.067	0.234	0.325	0.021
LVSI	Absent	17	2.0 (1.8–2.2)	0.8 (0.8–0.9)	39.4 (28.5–49.0)	6.9 (3.9–8.4)	7.4 (4.2–8.7)	0.1 (0.1—0.2)	23.0 (7.7–24.0)
	Present	3	1.9 (1.6–2.2)	0.8 (0.8–1.0)	49.1 (16.2–52.9)	5.6 (3.9–7.1)	5.2 (4.8–8.0)	0.2 (0.1–0.2)	13.7 (10.8–24.0)
	*p*		0.670	0.047	0.852	0.519	0.095	0.129	0.442

SUVmax: Standardized Uptake Value maximum; SCC: squamous cell carcinoma; LN: lymph node; LVSI: lymphovascular space invasion; PI: pulsatility index; RI: resistance index; PS: peak systolic velocity; ED: end-diastolic velocity; TAMAX: time-averaged maximum velocity. Following Benjamini–Hochberg correction for multiple testing across the 56 comparisons in this table, associations with PI (SUVmax, tumor volume, parametrial involvement, stage, lymph node involvement) and the correlation between tumor volume and RI or ED/PS remained statistically significant; associations involving RI or PS/ED with parametrial and lymph node status, and the histologic type comparisons (PI and TAMAX), did not survive correction and should be interpreted as exploratory.

The non-SCC group (*n* = 9) comprised four cases of cervical adenocarcinoma, two cases of adenocarcinoma in situ (AIS, one with microinvasion at FIGO stage IA1), one endocervical adenocarcinoma, one adenosquamous carcinoma, and one case of squamous cell carcinoma in situ.

After adjustment for age and menopausal status in multivariable linear regression, disease group remained a strong independent predictor of both PI and RI (Table 5). When the primary group comparison was repeated in premenopausal patients only (*n* = 131), the progressive increase in PI and RI with disease severity remained highly significant (Kruskal–Wallis *p* < 0.001 for both).

**Figure 2:**
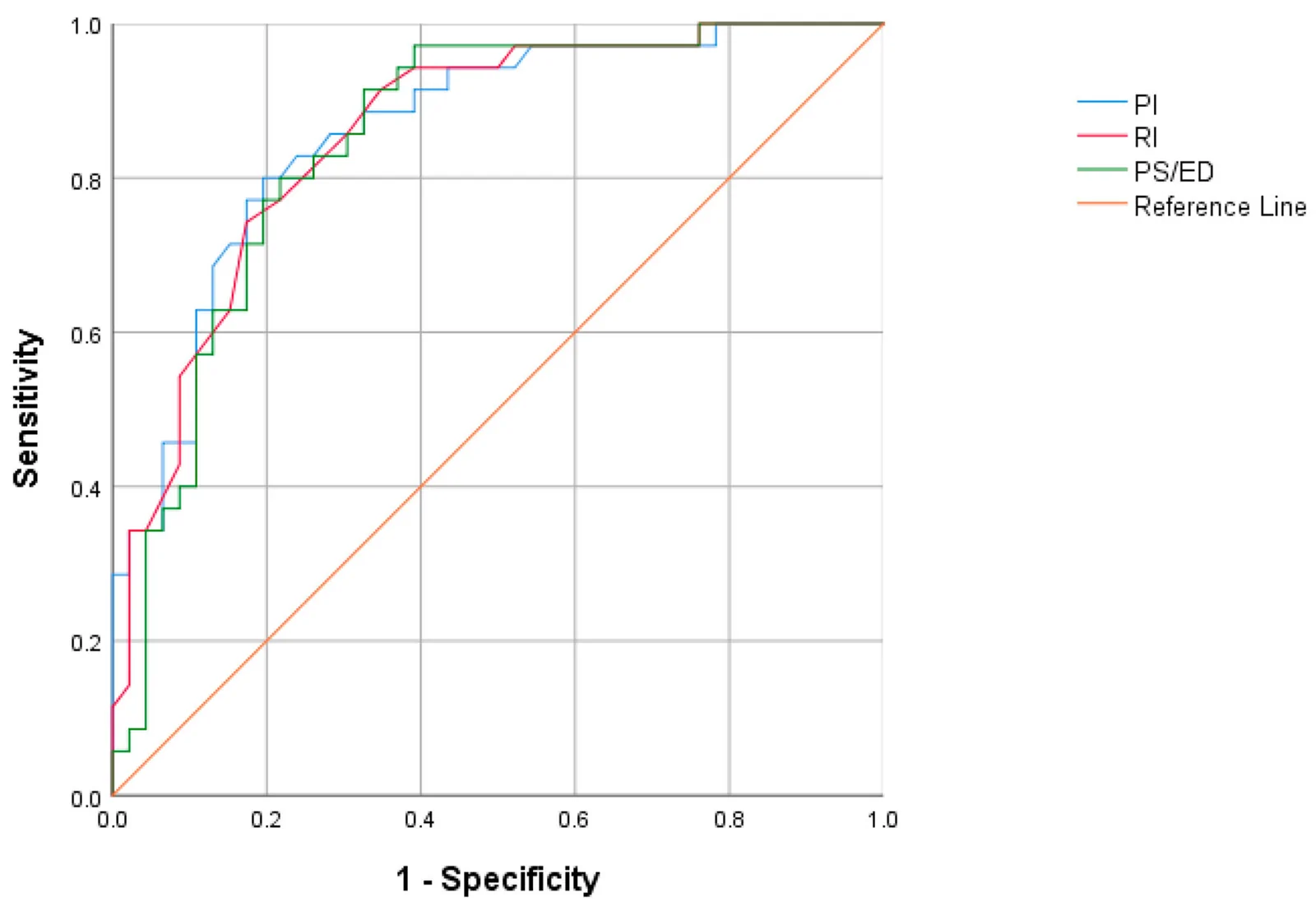
Receiver operating characteristic (ROC) curves for pulsatility index (PI), resistive index (RI), and peak systolic velocity/end-diastolic velocity ratio (PS/ED) in the discrimination of cervical cancer from healthy controls. The diagonal reference line represents chance-level discrimination (AUC = 0.50).

The ROC curve analysis revealed strong diagnostic efficacy for all three Doppler measures in distinguishing cervical cancer from healthy controls (Table 6). The AUC values were 0.861 (95% CI: 0.781–0.941) for PI, 0.857 (95% CI: 0.776–0.938) for RI, and 0.847 (95% CI: 0.762–0.933) for PS/ED, all reaching statistical significance (*p* < 0.001) (Figure 2). Among the three parameters, PI demonstrated the highest discriminatory capacity. At the optimal cutoff of 1.81, PI demonstrated a sensitivity of 80.0% and a specificity of 80.4%. The corresponding values for RI (cutoff: 0.81) were 74.3% and 82.6%, and for PS/ED (cutoff: 4.55) were 91.4% and 67.4%, respectively.

**Figure 3:**
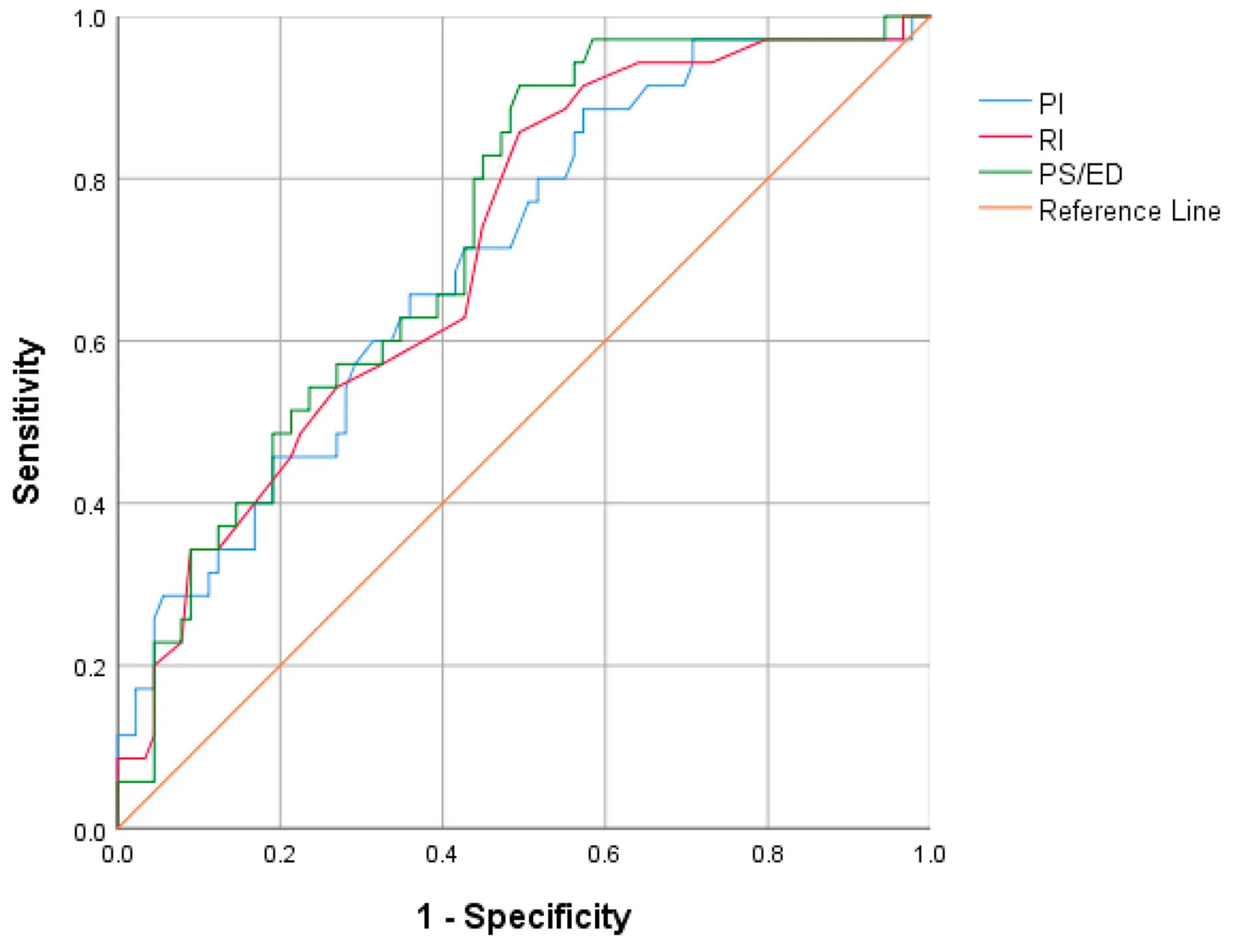
Receiver operating characteristic (ROC) curves for pulsatility index (PI), resistivity index (RI), and peak systolic velocity/end-diastolic velocity ratio (PS/ED) in the discrimination of cervical cancer from cervical intraepithelial neoplasia.

The ROC curve analysis indicated acceptable diagnostic efficacy for all three Doppler measures in distinguishing cervical cancer from CIN (Table 7). The AUC values were 0.704 (95% CI: 0.604–0.803) for PI, 0.712 (95% CI: 0.615–0.809) for RI, and 0.732 (95% CI: 0.639–0.824) for PS/ED, all achieving statistical significance (*p* < 0.001) (Figure 3). Of the three metrics, PS/ED had the greatest discriminating capability. At the ideal threshold of 4.63, PS/ED demonstrated a sensitivity of 91.4% and a specificity of 50.6%. The corresponding values for RI (cutoff: 0.79) were 85.7% and 50.6%, and for PI (cutoff: 1.69) were 88.6% and 42.7%, respectively.

Because CIN 1 represents low-grade disease with markedly lower PI and RI than CIN 2-3, its inclusion in the CIN comparator group may inflate apparent discrimination. When the comparison was restricted to cervical cancer versus CIN 2-3 only, the clinically most relevant distinction, discriminatory performance was substantially weaker (PI AUC 0.593, RI AUC 0.600, PS/ED AUC 0.622), and the difference in PI and RI did not reach statistical significance (*p* = 0.157 and *p* = 0.124, respectively).

## 4. Discussion

### 4.1. Summary of Main Results

In this prospective study, we evaluated patients diagnosed with CC and CIN by utilizing TVUSG to assess the significance of the UA Doppler indices across the groups. We found that increasingly elevated PI and RI were detected in the high-grade CIN and cervical cancer cohort in comparison to low-grade lesions, corresponding to decreased ED.

The findings of the present study were further investigated through two sub-analyses. In an exploratory subgroup analysis of patients who underwent LEEP for an initial diagnosis of CIN 3, PI was nominally higher among those with confirmed high-grade disease on final pathology, although this association did not remain significant after correction for multiple testing. Our results also showed that lesion volume was significantly associated with PI, RI, and PS/ED ratio. Additionally, the increased PI values were substantially correlated with well-known unfavorable clinicopathological factors, such as greater tumor volume, LN metastases, parametrial involvement, and advanced-stage disease.

### 4.2. Results in the Context of Published Literature

Tumor angiogenesis is a crucial hallmark of malignant progression and is fundamental during tumor growth, invasion, and metastatic spread, while the tumor microenvironment is also profoundly affected by the alteration of pro-angiogenic factors. As cervical lesions progress from low-grade CIN to invasive cancer, the hypoxic tumor microenvironment specifically promotes pro-angiogenic signaling pathways via Hypoxia-Inducible Factor 1-alpha (HIF-1α), leading to especially elevated Vascular Endothelial Growth Factor (VEGF), Transforming Growth Factor-beta (TGF-β) and Fibroblast Growth Factor-beta (FGF-β) expression. This biological cascade not only accelerates tumor cell proliferation but also boosts vascular permeability and stimulates the proliferation and migration of endothelial cells—producing the development of structurally aberrant vascular connections. Tumor vasculature is usually characterized by irregularity, fragility, high permeability, dilation and disorganization. Overall, the newly formed vascular network is recognized by the lack of smooth muscle support, the existence of arteriovenous shunts, and irregular diameter, which causes intratumoral heterogeneous perfusion, altered flow dynamics, microthrombosis/necrosis and increased interstitial pressure locally.

The most striking finding of this study, the progressive rise in uterine artery PI and RI with increasing lesion severity accompanied by a decline in ED, appears at first glance to contradict the classical low-resistance pattern described for tumor neoangiogenesis. This apparent discrepancy is most plausibly explained by the anatomical level at which Doppler sampling was performed. In the present study, measurements were obtained from the main trunk of the uterine artery at the isthmic level, proximal to its branching into the ascending and descending vessels, rather than from intratumoral or cervical branch vessels. Arterial Doppler indices at this proximal level reflect the aggregate impedance of the entire downstream vascular bed rather than local microvascular neoangiogenesis alone. Neoangiogenesis occurring within the tumor may generate low-resistance microvascular shunts that increase local blood flow, yet this does not necessarily translate into reduced impedance at the level of the feeding artery; conversely, tumor growth, extracellular matrix remodeling, and increased interstitial pressure within the cervical stroma can raise distal vascular impedance, reducing diastolic flow and consequently increasing PI and RI proximally. End-diastolic flow is particularly sensitive to this distal impedance, since it depends on the continuity of flow through the downstream vascular bed during diastole rather than on the systolic ejection pressure that largely determines peak systolic velocity; a rise in distal resistance therefore widens the difference between systolic and diastolic velocity, elevating both pulsatility and resistance indices. Most previous studies reporting the classical low-resistance pattern sampled intratumoral vessels, cervical branch arteries, or used transabdominal rather than transvaginal approaches, all of which more directly reflect local tumor neoangiogenesis. The divergence between the present findings and this body of literature therefore likely reflects differences in the vascular compartment examined rather than a genuine biological contradiction, and uterine artery Doppler findings should not be expected to mirror intratumoral Doppler findings directly. It should be noted that this mechanism, centered on tumor burden, stromal remodeling, and solid stress, applies specifically to invasive disease and cannot readily explain the marked increase in PI and RI already observed at the CIN 2-3 stage, which by definition remains confined to the epithelium without stromal invasion. The largest single increment in this cohort occurred between control and CIN 2-3 (PI: 1.49 to 2.14) rather than between CIN 2-3 and cancer (PI: 2.14 to 2.38), suggesting that a distinct, pre-invasive mechanism, potentially related to early angiogenic activation, altered vascular tone, or HPV-associated endothelial changes without accompanying mass effect, may underlie the pre-invasive gradient. This aspect of our findings is not explained by the invasion-dependent model proposed above and warrants dedicated investigation.

Transvaginal Doppler ultrasonography is a reliable, non-invasive, and effective tool to scan intratumoral blood flow with no cost and Doppler measurements may indirectly reflect the underlying root of angiogenic activity. A study by Doğan and colleagues [11] compared patients with or without HPV using transvaginal UA and cervical artery (CA) Doppler parameters. Both the RI of UA and CA were significantly higher in HPV-positive patients in comparison with the control group (*p* = 0.02 and *p* = 0.03, respectively), unlike PI values. Furthermore, the diagnostic efficacy of UA and CA vascularity indices, either independently or in conjunction with HPV DNA testing and cytology, were examined [9,11]. Consequently, they noted that the integration of CA-RI with hr-HPV or cytology diminished sensitivity while enhancing specificity [9,11]. The combination of UA-PI with hr-HPV significantly enhanced the positive predictive value compared to the hr-HPV test by itself [9,11]. Unfortunately, neither the UA nor the CA Doppler measurements were conclusive in differentiating CIN 1 or more advanced lesions at the beginning [11,24]. Similarly, in the prospective research conducted by Dağgez and colleagues [24], which had 225 patients, the UA Doppler parameters of the CIN 1, CIN 2-3, and control groups were analyzed. Neither the RI nor the PI values exhibited a statistically significant disparity across the groups (all *p* > 0.05) [24]. Conversely, Çakır and colleagues [9] assessed 45 hr-HPV types, 45 smear results and their related colposcopic biopsy results with regard to iliac artery (IA), UA and CA Doppler indices abdominally. There was no association between PI and RI of all three arteries and hr-HPV types and cytology, while the grade of CIN increased, CA-PI was found to be considerably diminished as a result of greater vascularization (CA-PI: 1.61 ± 0.43 for CIN 1 vs. 1.15 ± 0.28 for CIN 2-3, *p* = 0.038). Interestingly, in our analysis, CIN 2+ lesions were associated with higher UA-PI and UA-RI compared to others (all *p* = 0.001). Additionally, CIN 2+ lesions had remarkably lower ED than the control group (6 vs. 10, *p* = 0.001, respectively). However, there were no marked differences calculated for PS and TAMAX across groups. On the other hand, a retrospective study by Wu and colleagues [31] evaluated 130 patients diagnosed with pathology-proven high-grade CIN. The most significant blood flow color signals and the most prominent hotspots within the cervix were determined by TVUSG [31]. The results showed that the PI and RI values dramatically decreased from controls to high-grade CIN (all *p* < 0.001).

Another endpoint was to investigate the neovascularization process whether there was any difference between CIN 2-3 and invasive cancer. According to our findings, the study revealed no significant difference in Doppler indices between CC and CIN 2-3. This finding may be explained by the fact that vascular changes in CIN 3 are already advanced, making it difficult to detect a further hemodynamic shift at the point of invasion using UA measurements alone. Therefore, although TVDUSG may reliably distinguish low-grade from high-grade cervical disease, it appears less capable of differentiating CIN 2-3 from invasive cancer and should be used alongside other clinical and pathological assessments. In line with our findings, Tepper and colleagues [32] recorded transvaginal Doppler measurements within the cervical canal and concluded that the RI was not significantly different between patients with CC and patients with CIN 3 (*p* = 0.067).

Examining research on UA Doppler in cervical cancer, Garba and colleagues [19] evaluated 81 patients with cervical cancer abdominally and concluded that ED value was considerably higher in the CC group than in healthy controls (17.5 ± 6.08 vs. 7.53 ± 3.01, *p* < 0.0001), which reflected reduced PI and RI values, consistent with the classical high-flow, low-resistance pattern of tumor neoangiogenesis, in line with previous results [32]. Similarly, Zaria and colleagues [16] and Dağgez and colleagues [24] examined the uterine artery itself, the same vessel level assessed here, yet reported divergent findings: Zaria and colleagues observed a lower RI and higher PS in advanced-stage disease, consistent with the classical low-resistance pattern, while Dağgez and colleagues found no significant difference in PI or RI across CIN grades using a transvaginal approach. These uterine-artery-level discrepancies are not fully accounted for by the anatomical compartment hypothesis outlined above, since all three studies, including the present one, sampled the same vessel. Technical differences in transabdominal versus transvaginal insonation angle and depth resolution may partly explain the divergence from Garba and colleagues and Zaria and colleagues, both of whom used a transabdominal approach, while the null result of Dağgez and colleagues may reflect a smaller, differently distributed CIN cohort or a different segment of the uterine artery. We acknowledge that the compartment hypothesis, while consistent with the intratumoral literature, does not fully resolve these uterine-artery-level discrepancies, and further comparative studies using standardized sampling protocols are needed. On the other hand, the present study showed a disagreement with these results. We demonstrated that the UA-RI and UA-PI values were significantly elevated, and ED values were significantly reduced, in cervical cancer compared to controls. In this context, a case–control study conducted by Dodampahala and colleagues [13] measuring PI in the descending branch of the uterine artery, reported a mean PI of 1.94 in cervical carcinoma compared with 0.805 in controls, with values in invasive cancer reaching 1.99 to 3.10 versus 0.3 to 1.5 in controls, consistent with the direction of the present findings. Using ROC analysis, the authors identified an optimal PI cutoff of 1.475 for distinguishing malignancy from normal cervix, with 89.5% sensitivity and 93.5% specificity (AUC = 0.946), and concluded that PI may serve as a useful non-invasive marker of cervical malignancy [13].

In our subgroup analysis based on FIGO staging, a significantly higher PI was observed in patients with advanced-stage disease than those with early-stage disease, whereas no significant differences were observed in the other Doppler parameters. These results differ from those of Zaria and colleagues [16], who reported a significantly lower mean RI and higher PS values in advanced-stage disease compared to early-stage disease, consistent with the classical low-resistance pattern of tumor neoangiogenesis. However, several methodological differences may account for this discrepancy. Zaria and colleagues used transabdominal rather than transvaginal Doppler imaging, which differs in depth resolution and vascular visualization. Furthermore, their cohort included both pre- and postmenopausal patients without subgroup adjustment, and the authors themselves noted that menopausal status significantly influenced Doppler indices. These factors, combined with differences in disease stage distribution and patient demographics between the two populations, likely contributed to the contrasting hemodynamic patterns observed.

In addition to the FIGO stage, prognostic factors affecting survival and recurrence in cervical cancer patients include depth of stromal invasion, LVSI, tumor size, parametrial involvement, and lymph node metastasis. Recent research findings demonstrated that UA Doppler indices, specifically PI and RI, may have the potential to predict not just angiogenesis, but also the aggressiveness and metastatic potential of cervical malignancies. In this regard, numerous studies have remarked that the Doppler parameters have correlations with some adverse prognostic factors; however, the previous trials have revealed conflicting results [12,14,15,16,21].

A retrospective cohort study by Bolla and colleagues [14] identified 25 patients with CC undergoing assessment of Doppler indices of the UA before primary treatment. The results showed that a notable positive correlation was identified between ED and PS values and tumor size; however, no relation was detected for RI and PI. Likewise, Zaria and colleagues [16] revealed that the tumor volume had a significantly positive correlation with PS and ED, whereas a significantly inverse correlation with RI and PI. Relevant data from Spain assessed 27 patients with pathology-proven early-stage CC [12]. The amount of central intratumoral vessels was located by TVUSG and categorized as moderate or abundant. The abundant vascularization was notably related to LN metastasis, LVSI, deep stromal invasion, tumor size and parametrial involvement. They concluded that especially if the PI value within abundant vascularization was <0.82, adjuvant treatment was more likely to be needed. Tomalczyk and colleagues [15] offered an alternative perspective by enrolling 36 patients with locally advanced-stage cervical cancer and assessing transvaginal Doppler parameters in infiltrated and non-infiltrated uterine arteries depending on the parametrial involvement seen on magnetic resonance imaging (MRI) before treatment. In the first examination, the authors demonstrated that the median PS of infiltrated uterine arteries was higher than that of non-infiltrated ones (30.6 cm/s vs. 16.8 cm/s, respectively, *p* = 0.001).

These findings are not consistent with those of the present study. Elevated UA-PI values were substantially correlated with LN metastasis, increased SUVmax, SCC histology, parametrial invasion, and larger tumor volume. In contrast, LVSI positivity was not associated with PI but was associated with a significant difference in RI. With the higher PI and RI values in mind, an important clinical consideration is how the Doppler indices in the current results differ from those in the previous studies. Our research highlights the idea that the progressive tumor load may be linked with substantial vascular remodeling and circulatory instability. Moreover, from a biological point of view, aggressive cervical tumors exhibit high levels of angiogenic activity, stromal invasion, hypoxia-induced vascular disorganization, increased interstitial pressure, and the pressure of tumor mass, all of which may result in downstream vascular resistance detectable with Doppler ultrasonography. In addition, there is a possibility of disparity in both the peripheral and central vascularization of the tumor. Of note, the diameters of the vessels feeding the tumor may vary, and microthrombosis could develop in small-diameter vessels, resulting in hypoperfusion or necrosis in some areas of the tumor. This enhances angiogenic activation due to the elevation of HIF-1α, initiating the cycle.

The ROC analysis showed that all three Doppler parameters had diagnostic value in distinguishing cervical cancer from healthy controls. Among them, PI had the highest overall discriminatory performance. Clinically, PI showed a relatively balanced sensitivity and specificity at the optimal cutoff value. Therefore, it may be the most useful single Doppler parameter for preoperative assessment and could be considered as part of routine transvaginal ultrasonographic evaluation. This aligns with the findings of Dodampahala and colleagues [13] discussed above, who similarly identified PI as the most informative single Doppler parameter for distinguishing cervical malignancy, using a comparably high AUC (0.946). Consistent with this anatomical compartment explanation, Ye and colleagues recently reported that lower intratumoral PI and RI were associated with lymph node metastasis in cervical cancer, reflecting the classical low-resistance pattern at the intratumoral level.

The RI value showed the highest specificity among the three parameters. This suggests that RI may be more useful as a supportive or confirmatory parameter rather than as a primary screening marker. In contrast, PS/ED showed very high sensitivity in both comparisons, including cervical cancer versus healthy controls and cervical cancer versus CIN. This finding suggests that PS/ED may be helpful as a rule-out parameter across the cervical disease spectrum. However, its lower specificity limits its use as a standalone diagnostic marker. Similarly, Tepper and colleagues [32] investigated the most accurate RI’s cutoff values and reported that the best AUC test was 0.57 alongside a sensitivity of 81.0% and a specificity of 93.0%. Another notable result was revealed by Jurado and colleagues [12]. They analyzed the ROC curves for forecasting adjuvant therapy and determined that the optimal cutoff values for tumor diameter and deep stromal invasion were 17.5 mm (AUC: 0.66, 95% CI: 0.41–0.91) and 10 mm (AUC: 0.78, 95% CI: 0.57–0.98).

In addition to the previous studies, Wu and colleagues [31] further revealed the discrimination of high-grade CIN; at a PI threshold of 1.03, the ROC curves exhibited a sensitivity of 0.887, specificity of 0.838, and an AUC of 0.95. The study also showed that at a RI cutoff of 0.68, the sensitivity was 0.968, the specificity was 0.615, and the AUC was 0.84.

When the analysis was extended to the more difficult comparison between cervical cancer and CIN, the diagnostic performance of all parameters decreased. This decrease is likely related to the hemodynamic overlap between high-grade pre-invasive lesions and invasive cervical cancer, as discussed above. These findings suggest that no single Doppler parameter should be interpreted alone. Instead, a multiparametric approach including PI, RI, and PS/ED may provide more useful clinical information than any single parameter.

The comparatively low specificity of PI in distinguishing cervical cancer from CIN (42.7%) indicates that, unlike its performance against healthy controls, PI alone cannot reliably rule in malignancy within the high-grade pre-invasive to invasive disease continuum; a substantial proportion of high-grade CIN cases would be misclassified as cancer at the derived cutoff. Bootstrap validation further indicated that this apparent sensitivity and specificity are subject to meaningful optimism, with corrected estimates of 70.2% sensitivity and 57.8% specificity. In practice, this suggests that PI is best applied as a component of a broader diagnostic and staging pathway, alongside colposcopy, biopsy, and where indicated, cross-sectional imaging, rather than as a standalone triage test at the CIN to cancer transition. This limitation is further underscored when CIN 1 is excluded from the comparator group: PI, RI, and PS/ED all show markedly weaker discrimination between cervical cancer and CIN 2-3 specifically (AUC 0.59 to 0.62), the clinically most relevant distinction, with PI and RI failing to reach statistical significance. This indicates that the modest performance reported for the pooled CIN 1 to CIN 2-3 comparator group substantially overstates the test’s ability to distinguish cancer from its immediate precursor lesion.

To our knowledge, ROC-based studies evaluating these Doppler parameters across the full cervical disease spectrum are limited. Therefore, the cutoff values reported in the present study should be considered preliminary and need to be validated in larger, multicenter studies.

Aside from angiogenesis, another possible contributing mechanism is perivascular stromal and extracellular matrix remodeling. As the cervical neoplastic transformation progresses, the interaction of tumor cells with the adjacent stromal environment escalates, resulting in fibrosis, modified interstitial pressure, and vascular compression. These alterations may also enhance resistance in UA blood flow. As the neoplasia progressed, the vascular remodeling phase became predominant due to heterogeneous perfusion and persistent hypoxic areas within the tumor microenvironment. Increased stromal rigidity, interstitial pressure, perivascular compression, and disorganized immature microvessels may impair downstream perfusion and reduce diastolic flow velocities prior to the onset of substantial neoangiogenesis and arteriovenous shunting, consequently leading to increased PI and RI values on Doppler ultrasonography.

TAMAX reflects the mean of the maximum velocity envelope across the entire cardiac cycle rather than a single time-point measurement such as peak systolic or end-diastolic velocity, and therefore represents overall perfusion capacity and sustained flow velocity rather than vascular resistance per se. In contrast, PI and RI are determined primarily by waveform shape and are particularly sensitive to reductions in diastolic flow, which is typically the first parameter to decline as distal vascular resistance increases while systolic velocity remains relatively preserved. Because the uterine artery is a large conductance vessel, increases in distal microvascular resistance tend to alter the pulsatility of flow more than the mean maximum flow velocity itself, particularly at intermediate stages of disease. This physiological distinction is consistent with the pattern observed in the present study, in which PI and RI increased and end-diastolic velocity declined progressively with disease severity, while TAMAX remained essentially unchanged, suggesting that the observed hemodynamic alterations predominantly affect vascular impedance and diastolic flow rather than the overall maximum flow velocity sustained across the cardiac cycle.

Several factors may account for discrepancies in the Doppler indices between our findings and those in the published literature, potentially arising from variations in demographics and methodologies among studies. Initially, research groups frequently exhibit substantial distinctions in terms of lesion severity, HPV status, menopausal status, inflammatory background and the inclusion of invasive versus pre-invasive disease, all of which may affect cervical vascular hemodynamics. One of the key challenges to understanding the substantial variability of Doppler indices is the anatomical location of the ultrasound application, such as the UA, branch of the UA, or intracervical vessels. Of note, the Doppler values may evolve based on the ultrasonographic technique utilized, since transabdominal and transvaginal assessments vary in depth resolution and vascular imaging capabilities and may have different examination protocols, such as unilateral versus bilateral sampling strategies (recording mean values).

Lastly, in recent years, there have been several studies investigating the integration of different translational biomarkers or angiography models with ultrasound to improve the diagnostic accuracy and biological characterization of cervical neoplastic lesions. For instance, Zhu and colleagues [2] enrolled 235 patients with CC, high-grade CIN and controls and showed that quantitative superb microvascular imaging (SMI) allowed the detailed visualization of microvascular architectural field, thereby achieving improved diagnostic performance in distinguishing between pre-malignant and malignant lesions. Similarly, a retrospective cohort study reported that the use of contrast-enhanced ultrasound (CEUS) could serve as an indicator of malignancy, yet qualitative CEUS may not clearly separate all pre-malignant cervical lesions on its own [18]. The research by Wang and colleagues [33] demonstrated that the combination of serum CXCL16 and E-cadherin levels and CD ultrasound may offer further insights for the biological characterization and higher diagnostic sensitivity of cervical cancer.

Because the cervical cancer group was significantly older and more frequently postmenopausal than the control group, age and menopausal status were considered as potential confounders of the observed PI and RI gradient. Multivariable regression showed no independent effect of age, a modest independent effect of menopausal status on PI alone, and a persistently strong and independent effect of disease group on both indices; the gradient also remained significant when the analysis was restricted to premenopausal patients. These findings suggest that the observed Doppler changes are driven predominantly by disease severity rather than by age-related vascular stiffening or menopausal hormonal changes, although a residual confounding effect on PI specifically cannot be entirely excluded.

### 4.3. Strengths and Weaknesses

The main strength of the present study is its well structured prospective design, which mitigates recall and selection bias while enhancing the reliability of the results. The incorporation of a clearly delineated spectrum of cervical pathology—from normal controls to CIN 1, CIN 2–3, and invasive cervical cancer—facilitated a thorough assessment of alterations in Doppler parameters throughout the entire disease continuum, a characteristic that is often constrained in numerous prior studies. In addition, all uterine artery Doppler ultrasonography parameters were measured by a single examiner, which reduces interobserver variability and enhances measurement consistency. In addition, a more comprehensive and comparative analysis of hemodynamic parameters was performed by the simultaneous evaluation of multiple Doppler indices (PI, RI, PS, ED, PS/ED, ED/PS, TAMAX).

Furthermore, the study provides a ROC-based diagnostic performance analysis, demonstrating a notably strong discriminative feature, particularly for PI, hence enhancing the clinical relevance of the findings. This study’s strength also lies in the novel investigation of Doppler indices in relation to lesion volume following LEEP for high-grade CIN, an area insufficiently explored in the literature to our knowledge.

Several limitations of this study must be acknowledged. First, the data were collected at a single tertiary center, which would restrict the findings’ applicability to larger groups with various clinical or demographic features. Second, the relatively small sample size and limited number of events for each group may limit the statistical power. Third, the study lacked histopathological and molecular signs of angiogenesis, such as microvessel density or VEGF expression, which may have offered more robust biological support for the identified hemodynamic alterations. It should be acknowledged that HPV status in the control group was largely inferred from documented negative screening history rather than confirmed by concurrent testing in all cases, and that colposcopic referral in the CIN and cancer groups was itself often triggered by abnormal cytology, which correlates with HPV positivity. The comparison of HPV status across groups should therefore be interpreted with some circularity in mind, given that group definitions and HPV ascertainment were not fully independent, as is common practice in similar case–control designs. Side selection for uterine artery Doppler measurement was based on optimal visualization of the arterial trace rather than a fixed laterality protocol; contralateral measurement was required in an estimated maximum of 20 cases, though this was not systematically recorded during the study. Menstrual cycle phase was not standardized or systematically recorded for the 131 premenopausal patients in this study. Although no consensus guideline currently mandates a specific cycle phase for uterine artery Doppler assessment, cycle-related hormonal fluctuations may still contribute some additional variability to Doppler indices, representing a potential, if unquantified, source of variability not accounted for in the present analysis. This methodological difference, among others, may also contribute to the divergence between our findings and those of Dağgez and colleague [24], who elected to standardize measurements to the mid-luteal phase in their own protocol. The control group was itself partly defined by normal findings on transvaginal ultrasound, and the same examiner performed grayscale morphological assessment, including tumor echogenicity, immediately before Doppler measurement in each patient. This raises the possibility of a structural circularity in the cervical cancer versus control comparison that cannot be fully excluded despite blinding to final histopathological diagnosis at the time of Doppler evaluation.

Additionally, several subgroup analyses, particularly those involving lymphovascular space invasion status and histologic subtype, were based on small numbers of surgically treated patients (*n* = 20), increasing the risk of type II error and limiting the precision of these estimates. Although false discovery rate correction was applied to the associations reported in Table 4, the exploratory nature of these secondary analyses should be kept in mind, and the findings that did not survive correction should be regarded as hypothesis-generating rather than confirmatory.

### 4.4. Implications for Practice and Future Research

The findings of this study have several practical implications. Transvaginal uterine artery Doppler ultrasonography is widely available, reproducible, and free of radiation exposure, making it a feasible adjunct to existing diagnostic pathways, especially in set-tings where MRI or PET-CT access is limited. The significant association between preoperative PI values and adverse clinicopathological features such as parametrial involvement, LN metastasis, and advanced-stage suggests that Doppler indices may support preoperative risk stratification and treatment planning in cervical cancer patients. Combining Doppler parameters with other diagnostic tools such as hr-HPV testing or quantitative vascular imaging may further improve diagnostic accuracy, as suggested by recent studies. Prospective validation in larger, multicenter cohorts is needed before these findings can be incorporated into routine clinical practice.

## 5. Conclusions

Transvaginal uterine artery Doppler ultrasonography demonstrated meaningful associations with cervical neoplastic severity across the full disease spectrum from healthy controls to invasive cervical cancer. PI and RI values increased progressively with disease severity, while ED declined, reflecting underlying hemodynamic changes associated with neoplastic progression. Among the evaluated parameters, PI showed the strongest diagnostic performance in distinguishing cervical cancer from healthy controls. Notably, discriminatory performance was substantially weaker when cervical cancer was compared specifically with CIN 2-3 rather than the pooled CIN group, indicating that Doppler indices are considerably less useful for distinguishing cancer from its immediate high-grade precursor than from healthy tissue. Elevated PI was also associated with adverse prognostic features including parametrial involvement, LN metastasis, larger tumor volume, and advanced FIGO stage. Given the exploratory nature of several subgroup findings and the absence of longitudinal outcome data, these associations should be regarded as hypothesis-generating pending validation in larger, independent cohorts. These findings suggest that Doppler ultrasonography may serve as a useful, non-invasive adjunct in the preoperative evaluation of cervical neoplasia. Future studies with larger cohorts, molecular angiogenesis markers, and survival endpoints are needed to confirm and extend these results.

## Figures and Tables

**Table 1:** Comparative analysis of clinical characteristics and treatment modalities in cervical intraepithelial neoplasia (CIN), cervical cancer and control patients.

Patient Characteristic	Control, *N* (%) Total = 46	CIN 1, *N* (%) Total = 43	CIN 2-3, *N* (%) Total = 46	Cervical Cancer, *N* (%) Total = 35	*p*-Value
Age	38.50 ± 7.41 ^a^	40.02 ± 10.05 ^a^	38.41± 9.84 ^a^	48.02± 12.79 ^b^	**0.001**
Menopause					**0.002**
Postmenopausal	6 (13.04%)	10 (23.26%)	7 (15.22%)	16 (45.71%)	
Premenopausal	40 (86.96%)	33 (76.74%)	39 (84.78%)	19 (54.29%)	
Gravida	2 (1–3)	2 (0–3)	1 (0–3)	2 (2–3)	0.065
Parity	2 (1–2) ^a^	1 (0–2) ^a^	1 (0–2) ^ab^	2 (1–3) ^ac^	**0.015**
Delivery					0.099
None	9 (19.57%)	13 (30.23%)	16 (34.78%)	3 (8.57%)	
C/S	22 (47.83%)	17 (39.53%)	14 (30.43%)	15 (42.86%)	
Vaginal	15 (32.61%)	13 (30.23%)	16 (34.78%)	17 (48.57%)	
HPV					**0.001**
Absent	20 (100.0%)	1 (2.86%)	1 (2.70%)	0 (0.0%)	
Present	0 (0.0%)	34 (97.14%)	36 (97.30%)	10 (100.0%)	
Symptom					**0.001**
Absent	46 (100.0%)	35 (81.40%)	40 (86.96%)	10 (28.57%)	
Present	0 (0.0%)	8 (18.60%)	6 (13.04%)	25 (71.43%)	
Smoking					**0.028**
Absent	36 (78.26%)	27 (62.79%)	29 (63.04%)	16 (45.71%)	
Present	10 (21.74%)	16 (37.21%)	17 (36.96%)	19 (54.29%)	
Primary Treatment					
Surgery	0 (0.0%)	0 (0.0%)	0 (0.0%)	20 (57.14%)	
Cryotherapy	0 (0.0%)	23 (53.49%)	0 (0.0%)	0 (0.0%)	
CRT	0 (0.0%)	0 (0.0%)	0 (0.0%)	15 (42.86%)	
LEEP	0 (0.0%)	0 (0.0%)	46 (100.0%)	0 (0.0%)	
Follow-up	46 (100.0%)	20 (46.51%)	0 (0.0%)	0 (0.0%)	

Data are presented as mean ± standard deviation, median (interquartile range), or *n* (%). CIN: cervical intraepithelial neoplasia; C/S: cesarean section; LEEP: loop electrosurgical excision procedure; CRT: chemoradiotherapy; HPV: human papillomavirus. Superscript letters indicate statistically significant differences between specific groups. The *p*-values were calculated using Chi-square test, Fisher’s exact test, Kruskal–Wallis test, or one-way ANOVA as appropriate.

**Table 2:** Vascular indices by color Doppler ultrasonography according to cervical disease severity.

Vascular Indices	Control	CIN 1	CIN 2-3	Cervical Cancer	*p*-Value
PI	1.49 ± 0.54 ^a^	1.68 ± 0.42 ^a^	2.14 ± 0.57 ^b^	2.38 ± 0.68 ^b^	0.001
RI	0.74 (0.64–0.79) ^a^	0.74 (0.72–0.79) ^a^	0.82 (0.76–0.87) ^b^	0.84 (0.80–0.89) ^b^	0.001
PS (cm/s)	36 (24–49)	27 (22–42)	36 (25–51)	41 (28–53)	0.096
ED (cm/s)	10 (7–13) ^a^	6 (5–10) ^a^	6 (4–10) ^ab^	5 (4–8) ^b^	0.001
PS/ED	4 (3–5) ^a^	4 (3–5) ^a^	6 (4–8) ^b^	7 (5–9) ^b^	0.001
ED/PS	0.29 (0.21–0.47) ^a^	0.26 (0.21–0.29) ^a^	0.17 (0.13–0.24) ^b^	0.15 (0.11–0.19) ^b^	0.001
TAMAX (cm/s)	15 (11–22)	12 (10–21)	14 (9–19)	13 (11–23)	0.797

Data are presented as mean ± standard deviation or median (interquartile range). CIN: cervical intraepithelial neoplasia; PI: pulsatility index; RI: resistance index; PS: peak systolic velocity; ED: end-diastolic velocity; TAMAX: time-averaged maximum velocity. ^a, b, c^ Groups not sharing a common superscript letter differed significantly at *p* < 0.05 following Bonferroni correction; groups sharing the same letter did not differ significantly. The *p*-values were calculated using Kruskal–Wallis test or one-way ANOVA as appropriate.

**Table 5:** Univariable and multivariable linear regression analyses of disease group, age, and menopausal status as predictors of uterine artery PI and RI.

Predictor	Univariable β (95% CI)—PI	*p*	Multivariable β (95% CI)—PI	*p*	Univariable β (95% CI)—RI	*p*	Multivariable β (95% CI)—RI	*p*
Disease group (ordinal)	0.315 (0.238–0.392)	<0.001	0.289 (0.211–0.367)	<0.001	0.060 (0.045–0.076)	<0.001	0.056 (0.040–0.072)	<0.001
Age (years)	0.017 (0.008- 0.026)	<0.001	−0.0004 (−0.012–0.011)	0.952	0.003 (0.001–0.005)	0.001	0.001 (−0.002–0.003)	0.546
Postmenopausal status	0.475 (0.250–0.700)	<0.001	0.319 (0.025–0.613)	0.034	0.074 (0.028–0.120)	0.002	0.029 (−0.032–0.089)	0.352

In a sensitivity analysis additionally adjusting for smoking status, the disease group effect remained materially unchanged (PI: β = 0.297, *p* < 0.001; RI: β = 0.057, *p* < 0.001), and smoking itself showed no independent association with PI or RI (*p* = 0.37 and *p* = 0.46, respectively). Variance inflation factors for all covariates in an indicator-coded (dummy variable) version of the model were below 2.4, indicating no meaningful collinearity between disease group and age or menopausal status; the interpretation above should therefore not be attributed to collinearity.

**Table 6:** Diagnostic performance of Doppler parameters in differentiating cervical cancer from healthy controls: ROC curve analysis.

Parameter	AUC	95% CI	*p*-Value	Optimal Cutoff	Sensitivity (%)	Bootstrap-Corrected Sensitivity (%)	Specificity (%)	Bootstrap-Corrected Specificity (%)
PI	0.861	0.781–0.941	<0.001	1.81	80.0	79.6	80.4	78.0
RI	0.857	0.776–0.938	<0.001	0.81	74.3	82.5	82.6	73.0
PS/ED	0.847	0.762–0.933	<0.001	4.55	91.4	85.3	67.4	71.6

AUC: area under the curve; CI: confidence interval; PI: pulsatility index; RI: resistive index; PS/ED: peak systolic velocity/end-diastolic velocity ratio. Optimal cutoff values were determined by the Youden index (sensitivity + specificity − 1). Bootstrap-corrected estimates (2000 resamples) reflect internal validation of the Youden-derived cutoff and provide a more conservative estimate of real-world diagnostic performance than the apparent values derived from the same dataset used to select the cutoff.

**Table 7:** Diagnostic performance of Doppler parameters (PI, RI, and PS/ED) in differentiating cervical cancer from cervical intraepithelial neoplasia: ROC curve analysis.

	Comparison	AUC	95% CI	*p*-Value	Optimal Cutoff	Sensitivity (%)	Bootstrap-Corrected Sensitivity (%)	Specificity (%)	Bootstrap-Corrected Specificity (%)
PI	CC vs. CIN 1–3 (pooled)	0.704	0.604–0.803	<0.001	1.69	88.6	70.2	42.7	57.8
PI	CC vs. CIN 2–3 only	0.593	0.469–0.713	0.157	2.90	28.6	54.5	89.1	59.8
RI	CC vs. CIN 1–3 (pooled)	0.712	0.615–0.809	<0.001	0.79	85.7	79.1	50.6	53.8
RI	CC vs. CIN 2–3 only	0.600	0.475–0.718	0.124	0.89	34.3	54.7	87.0	62.8
PS/ED	CC vs. CIN 1–3 (pooled)	0.732	0.639–0.824	<0.001	4.63	91.4	85.0	50.6	53.5
PS/ED	CC vs. CIN 2–3 only	0.622	0.502–0.748	0.061	4.65	91.4	68.6	32.6	52.0

AUC: area under the curve; CI: confidence interval; PI: pulsatility index; RI: resistive index; PS/ED: peak systolic velocity/end-diastolic velocity ratio. Optimal cutoff values were determined by the Youden index (sensitivity + specificity − 1). Bootstrap-corrected estimates (2000 resamples) reflect internal validation of the Youden-derived cutoff and provide a more conservative estimate of real-world diagnostic performance than the apparent values derived from the same dataset used to select the cutoff. Because CIN 1 represents low-grade disease with markedly lower PI and RI than CIN 2-3, its inclusion in the comparator group may inflate apparent discrimination. The CC versus CIN 2-3 comparison represents the clinically decisive distinction and is reported here for transparency; *p*-values for this comparison reflect the Mann–Whitney test of the underlying Doppler values between groups.

## Data Availability

Due to hospital policies, patient data and study materials cannot be shared. However, the data are available from the corresponding author upon reasonable request.

## Abbreviations

The following abbreviations are used in this manuscript:

AIS	Adenocarcinoma in situ
ANOVA	Analysis of variance
AUC	Area under curve
CA	Cervical artery
CC	Cervical cancer
CD	Color Doppler
CEUS	Contrast-enhanced ultrasound
CI	Confidence Interval
CIN	Cervical intraepithelial neoplasia
CRT	Chemoradiotherapy
CXCL16	C-X-C motif chemokine ligand 16
ED	End-diastolic velocity
FGF-β	Fibroblast Growth Factor-beta
FIGO	International Federation of Gynecology and Obstetrics
HIF-1α	Hypoxia-Inducible Factor 1-alpha
HPV	Human papillomavirus
hr-HPV	High-risk human papillomavirus
IA	Iliac artery
IQR	Interquartile range
LEEP	Loop electrosurgical excision procedure
LN	Lymph node
LVSI	Lymphovascular space invasion
MRI	Magnetic resonance imaging
PET-CT	Positron emission tomography/computed tomography
PD	Power Doppler
PI	Pulsatility index
PID	Pelvic inflammatory disease
PS	Peak systolic velocity
RI	Resistance index
ROC	Receiver Operating Characteristic
SCC	Squamous cell carcinoma
SMI	Superb microvascular imaging
SPSS	Statistical Package for the Social Sciences
SUVmax	Standardized Uptake Value maximum
TAMAX	Time-averaged maximum velocity
TGF-β	Transforming Growth Factor-beta
TVDUSG	Transvaginal Doppler ultrasonography
TVUSG	Transvaginal Ultrasonography
UA	Uterine artery
VEGF	Vascular Endothelial Growth Factor
